# Prolonged Use of Tirofiban Infusion Without Percutaneous Coronary Intervention To Achieve Optimal Results in a COVID-19 Positive Patient With Inferior ST Segment Elevated Myocardial Infarction (STEMI) Secondary to Thromboembolism: A Case Report

**DOI:** 10.7759/cureus.30287

**Published:** 2022-10-14

**Authors:** Zahid Khan, Niket Patel

**Affiliations:** 1 Acute Medicine, Mid and South Essex NHS Foundation Trust, Southend on Sea, GBR; 2 Cardiology and General Medicine, Barking, Havering and Redbridge University Hospitals NHS Trust, London, GBR; 3 Cardiology, Royal Free Hospital, London, GBR

**Keywords:** ultrasonic aspiration, sliding scale, non-peptidal antagonist of the gp iib/iiia receptor, tirofiban, thrombo embolic disease, electrocardiogram (ecg/ekg), diabetes type 2, covid 19 associated acute coronary syndrome, covid 19, st-elevation myocardial infarction (stemi)

## Abstract

Severe acute respiratory syndrome‑coronavirus‑2 (SARS‑CoV‑2), responsible for COVID-19, is mainly a respiratory illness, but it can affect other organs also such as heart, kidneys, and liver. Myocardial injury from COVID-19 has been reported in hospitalized patients ranging from pericarditis and myocarditis to acute coronary syndrome (ACS). COVID-19 is highly hypercoagulable state and is associated with both central and peripheral thromboembolism. COVID 19 patients with ACS may not present with classical features of chest pain and electrocardiogram (ECG) is the most important initial investigation in these patients to assess for any ST or T waves changes. COVID-19 patients with cardiac involvement are the most vulnerable group of patients and have increased morbidity and mortality risk. COVID-19 infections can affect the cardiovascular system in patients with or without history of coronary artery disease (CAD), but the risk of type 1 or 2 myocardial infarction (MI), myocardial injury, ST segment elevation, myocarditis, heart failure, cardiogenic shock, and life threatening arrhythmias are more common in the former group. We present a case of 55-year-old patient who presented to our cardiac center with ST elevated myocardial infarction and high blood sugar level. Patient was recently diagnosed with type 2 diabetes mellitus (T2DM) but was not commenced on medications. Echocardiogram showed mildly impaired left ventricular systolic function (LVSF) with inferior wall hypokinesia, and ECG showed inferior leads ST elevation. Coronary angiogram showed severe mid-vessel lesion and occluded posterior left ventricular branch (PLV). Multiple attempts at aspirating the thrombus resulted in thrombolysis in MI grade 2 (TIMI 2) flow in the vessel and patient was commenced on a tirofiban infusion for 72 hours.

## Introduction

Severe acute respiratory syndrome coronavirus 2 (SARS-CoV-2) mainly affects the respiratory system but can also affect cardiovascular system, gastrointestinal tract, and other systems. The coronaviruses belong to the Coronaviridae family, Orthocoronavirinae subfamily, and can tolerate very lower temperature that increases its virulence and pathogenicity [1]. Approximately 15% of COVID-19 patients develop septic shock due to pneumonia that requires hospitalization and about 5% of COVID-19 positive patients require ICU admission [2]. Patients with cardiovascular disease are at higher risk to develop serious complication from COVID-19 infection [3]. The presence of COVID-19 infection and coronary artery disease (CAD) is associated with higher morbidity and mortality due to acute coronary syndrome (ACS), arrhythmia, heart failure, thromboembolism, and cardiogenic shock [3]. The multisystem involvement of COVID-19 has caused significant additional challenges to treat them along with controlling the disease.

During the peak of COVID-19 last year, mortality from COVID-19-induced ACS was extremely high since patients attended hospitals very late or avoided coming to hospitals due to fear of catching the infection [4]. The extreme pressure on healthcare system exerted by COVID-19 infection compromised the standard and well-established treatment for ACS, including ST segment elevated myocardial infarction (STEMI) [4]. The typical ACS symptoms of chest pain with or without dyspnea are not that specific and sensitive anymore since COVID-19 patients also present with similar symptoms in most cases [5]. Occasionally, the ST segment elevation can be transient on electrocardiogram (ECG) suggestive of type 2 myocardial infarction (MI) due to severe hypoxia, acute inflammatory process, and respiratory failure [6]. Acute myocardial injury may occur due to plaque rupture, or type 2 MI due to oxygen supply and demand mismatch, myocardial injury to disseminated intravascular coagulation (DIC) or a non-ischaemic aetiology such as myocarditis, takotsubo cardiomyopathy, or direct myocardial invasion [6]. ACS, on the other hand, defines three characteristic clinical syndromes that include non-ST segment elevated myocardial infarction (NSTEMI), STEMI, and unstable angina. COVID-19 patients who developed ACS tend to have worse prognosis [6]. In addition to the usual cardiovascular risk factors for ACS, patients with COVID-19 have higher ACS risk due to prothrombotic activation of the coagulation cascade, endothelial dysfunction, cytokine-mediated systemic inflammatory response, and hypoxic injury due to oxygen supply/demand imbalance.

Most STEMI cases in COVID-19 patients were found to have no obvious coronary artery obstruction on coronary angiogram suggestive of MI with nonobstructive coronary arteries (MINOCA). We present a case of 55-year-old patient who presented with inferolateral STEMI on ECG and coronary angiogram showed severe mid-vessel right coronary artery (RCA) lesion and occluded posterior left ventricular branch (PLV). Multiple attempts were made to aspirate the clot and patient was commenced on a tirofiban infusion for STEMI pathway.

## Case presentation

**Figure 1:**
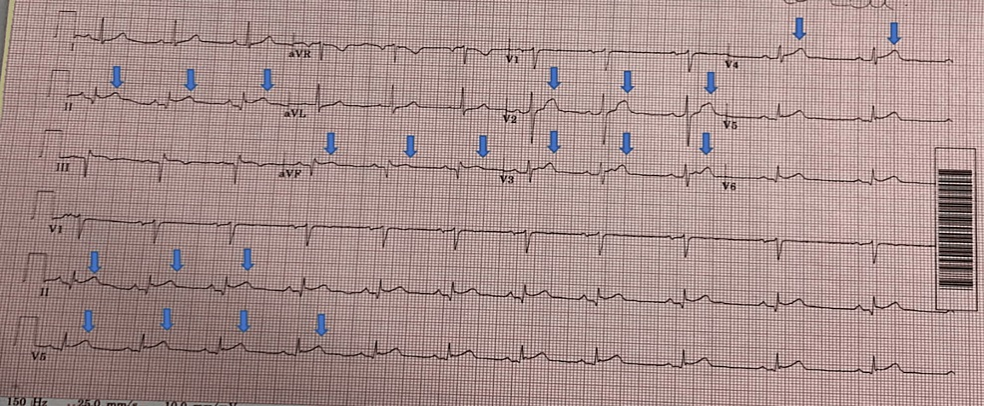
ECG shows ST segment elevation in infero-lateral leads ECG: Electrocardiogram

A 55-year-old patient presented with chest pain from 6 am in the morning, which was a dull, central chest pain and dyspnoea. His past medical history was relevant for psoriatic arthropathy, erectile dysfunction, poliomyelitis, and recently diagnosed type 2 diabetes mellitus (T2DM), but the patient was not commenced on any treatment so far. Regular medications included artificial saliva, and he also received intra-articular steroid injections a few times in the past for osteoarthritis. The last steroid injection was two months ago. He was a lifelong non-smoker and non-drinker. On arrival at the hospital, blood pressure was 174/96 mmHG, heart rate 70 bpm, respiratory rate 20 bpm, oxygen saturation 96% on room air, and temperature 36.7°C. Patient was loaded with 300 mg aspirin and 5 mg morphine by paramedics and was given 180 mg ticagrelor as a stat dose in the hospital. He denied any cough and calf pain. Patient was given glyceryl trinitrine spray. He had ongoing chest pain on arrival at the cardiac center. COVID-19 SARS CoV-2 RNA test was positive and laboratory tests results are shown in Table 1. ECG showed ST segment elevation in inferolateral leads (Figure 1) and bedside echocardiogram showed mildly impaired left ventricular systolic function (LVSF) with ejection fraction about 50% (Video 1-3). 

**Table 1:** Laboratory tests' results trend for patient

Test	Day 1	Day 2	Day 3	Reference value
White cell count	6.75	6.66	8.14	3.5-11 x 10^9^/L
Neutrophil	3.68	4.96	4.04	1.7-7.5 x 10^9^/L
Haemoglobin	163	148	135	135-170 g/L
Platelet	246	230	232	140-400 x 10^9^/L
Urea	2.6	2.3	2.2	2.9-8.2 mmol/L
Creatinine	47	35	36	66-112 umol/L
Sodium	135	133	132	135-145 mmol/L
Potassium	4.0	3.9	3.3	3.5-5.1 mmol/L
C-reactive protein	2	3	2	0-5 mg/L
Troponin	62	155	1685	< 14 ng/L
D-dimer	379	476	-	0-400 ng/mL
N-terminal pro-brain natriuretic peptide (pro-BNP)	50	173	-	< 400 ng/L
Bilirubin	7	7	8	0-21 umol/L
Alanine aminotransferase	54	47	54	10-50 unit/L
Aspartate aminotransferase	37	56	135	10-50 unit/L
Alkaline phosphatase	316	270	211	0-129 unit/L

**Video 1:** Echocardiogram apical 4 chambers view shows mild LVSD and inferior wall hypokinesia LVSD: Left ventricular systolic dysfunction

**Video 2:** Apical 4 chambers zoomed view shows mild LVSD and inferior wall hypokinesia LVSD: Left ventricular systolic dysfunction

**Video 3:** Echocardiogram 3 chambers view shows inferior wall hypokinesia and mild LVSD LVSD: Left ventricular systolic dysfunction

**Figure 2:**
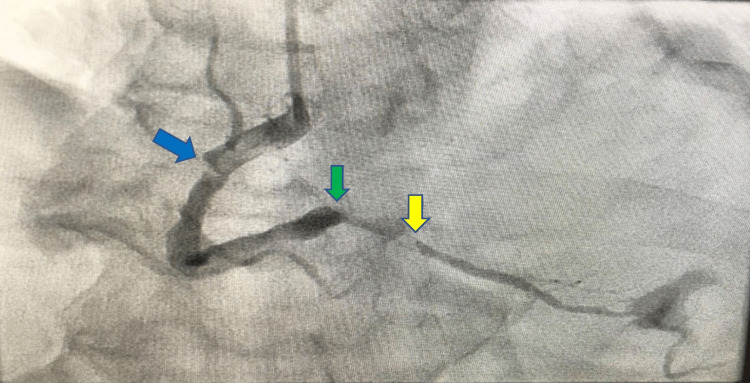
Coronary angiogram shows mid-vessel RCA lesion (blue arrow), occluded PLV (green arrow), and PDA lesion (yellow arrow) RCA: Right coronary artery; PLV: Posterior left ventricular branch; PDA: Posterior descending artery

**Figure 3:**
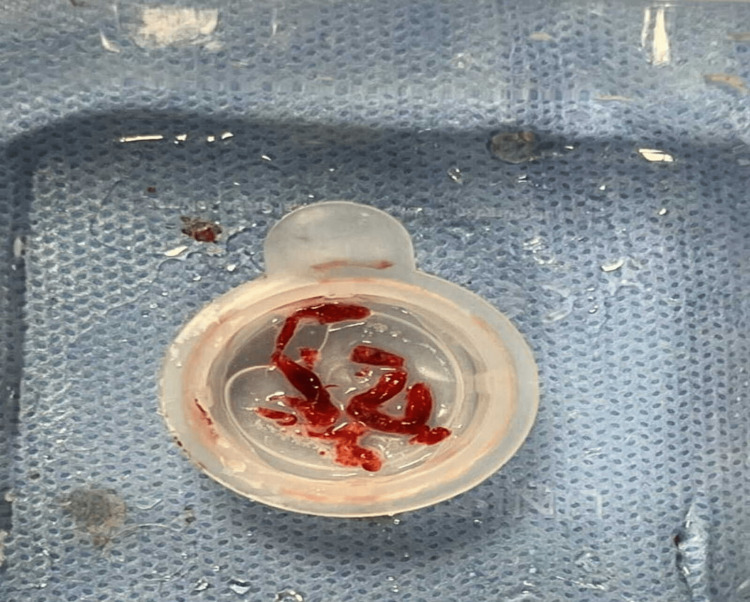
Thrombus aspirated from mid-vessel RCA, PLV, and PDA RCA: Right coronary artery; PLV: Posterior left ventricular branch; PDA: Posterior descending artery

Coronary angiogram showed sluggish flow in the left descending artery and severe mid vessel lesion in the RCA and occluded PLV. Thrombus aspiration was attempted, and several thrombi were aspirated by using aspiration catheter from RCA. We achieved thrombolysis in MI grade 2 (TIMI 2) flow in the RCA and PLV branch following thrombus aspiration. Following this, we dilated the mid vessel RCA lesion with 2.5 mm semi-compliant balloon, which resulted in occlusion of the PLV branch again. Further thrombus aspiration was attempted from the distal PLV branch resulting in TIMI 2 flow in it. Following this, thrombus aspiration was attempted from the proximal vessel, which resulted in occlusion of the posterior descending artery (PDA) and distal and proximal thrombus aspiration was attempted again achieving TIMI 2 flow (Videos 4-8, Figure 2). Figure 3 shows the thrombus aspirated from the RCA and its branches.

**Video 4:** Coronary angiogram shows mid vessel RCA stenosis and occluded PLV RCA: Right coronary artery; PLV: Posterior left ventricular branch

**Video 5:** Coronary angiogram shows normal LMS, LCx, and sluggish flow in the LAD LMS: Left main stem; LCx: Left circumflex artery; LAD: Left anterior descending artery

**Video 6:** Coronary angiogram shows mid-vessel RCA lesion and occluded PDA branch following balloon inflation of the mid- vessel RCA lesion RCA: Right coronary artery; PDA: Posterior descending artery

**Video 7:** Coronary angiogram shows mid-vessel RCA lesion, occluded PLV, and PDA lesion following attempted aspiration and ballooning of the mid-vessel RCA lesion RCA: Right coronary artery; PDA: Posterior descending artery; PLV: Posterior left ventricular branch

**Video 8:** Coronary angiogram shows TIMI 2 flow in RCA following thrombus aspiration in the RCA TIMI 2: Thrombolysis in myocardial infarction grade 2; RCA: Right coronary artery

 

**Figure 4:**
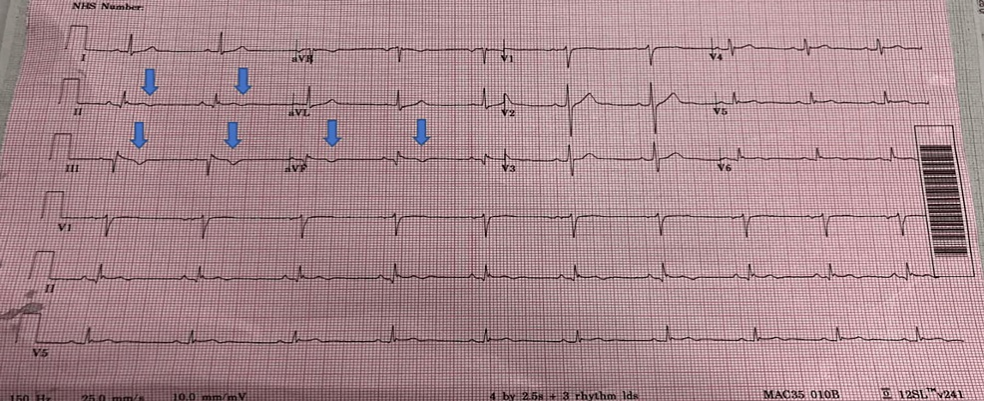
Post coronary angiogram ECG shows TWI in inferior leads (arrows) TWI: T waves inversion

He was commenced on STEMI protocol tirofiban infusion (250 ml) in 250 ml sodium chloride at a rate of 0.155 mcg/kg/min for the next 72 hours. He was also commenced on sliding scale as his blood sugars were 23.0. He was recently diagnosed with T2DM and his last glycated hemoglobin (HbA1c) two months ago was 96, but he was not commenced on any medication for his diabetes. Repeat HbA1c on this admission was 94. He had a repeat coronary angiogram 96 hours from the first angiogram that showed significant plaque resolution and restoration of TIMI 3 flow in RCA (Video 9). ECG showed T waves inversion (TWI) in inferior leads and normal sinus rhythm (Figure 4). He was continued with dual antiplatelet therapy (aspirin 75 mg once daily (OD) and ticagrelor 90 mg twice daily (BD)) for 12 months and rivaroxaban 20 mg OD for two months. He was also seen by diabetes specialist team and was commenced on NovoMix insulin. He remained pain free and was discharged home with outpatient follow up and repeat angiogram in three months.

**Video 9:** Coronary angiogram shows significant plaque resolution and TIMI 3 flow in RCA TIMI 3: Thrombolysis in myocardial infarction grade 3; RCA: Right coronary artery

 

## Discussion

COVID-19 infection has been reported to be associated with both arterial and venous thromboembolism (VTE) such as pulmonary embolism (PE), cerebrovascular accident (CVA), acute myocardial infarction (AMI), renal artery thrombosis, and mesenteric ischemia [7]. The exact pathophysiology for this increased thrombotic risk is unclear, although immune system hyperactivity with cytokine storm, local and systemic inflammatory reactions, and diffuse microthrombi are the possible mechanisms that contribute to this higher thromboembolic risk in COVID-19 patients [7,8]. Myocardial injury has been reported in up to 12% of hospitalized patients with COVID-19 and 20% patients requiring admission to ICU [9]. Other possible explanations for this increased thrombotic risk include angiotensin converting enzyme-2 induced endothelial cell and microvascular dysfunction or occlusion in the coronary arteries [9]. The pro-inflammatory state in COVID-19 patients may promote destabilization of a coronary atherosclerotic plaque leading to type 1 AMI and this phenomenon was previously observed in influenza pandemic [8]. COVID-19 infections can also cause other cardiovascular complications such as myocarditis, pericarditis in addition to type 1 and type 2 AMI [9].

Several case studies have reported the association between COVID-19 infection and AMI [8-10]. Saririan et al. published a case series of 3 patients with COVID-19 infection presenting with STEMI [9].They reported the incidence of myocardial injury in hospitalized patients with COVID-19 to range from 23% to 27.8%. The first patient in this case series was 61 years old, who presented with supraventricular tachycardia (SVT) who was successfully cardioverted with adenosine. However, ECG post cardioversion confirmed STEMI in anterolateral leads. The patient was treated for possible myopericarditis, and coronary angiogram at a later date did not show any CAD. The second patient was a 59-year-old patient presenting with shortness of breath and was intubated. ECG post intubation showed ST-segment elevations in V1-V4 and reciprocal ST-depressions in leads II, III, and aVF, and patient underwent coronary angiogram that showed moderate disease in the left main stem (LMS) only and the remaining coronary arteries were normal [9].

Lakhdar et al. published a case series of six patients with COVID-19 who either presented with STEMI or developed STEMI during hospital admission [1]. All these six patients had predominantly inferior territory ST segment elevation on ECGs and these patients were thrombolysed due to haemodynamic instability and none of these patients survived unfortunately. Isnanijah et al. published a case report of a 52-year-old patient presenting with abdominal pain and had cardiac arrest in the emergency department. Following return of spontaneous circulation (ROSC), ECG showed anterior STEMI and patient had primary percutaneous intervention (PCI) to left anterior descending artery (LAD) [4]. A study from England reported that most patients presenting with AMI during the pandemic lockdown were younger, non-diabetic, and less likely to have a prior cardiovascular or cerebrovascular disease [11]. It is also important to mention that overall patient’s presentation to hospitals with AMI declined during COVID-19 pandemic which is likely due to patient's reluctance to attend hospitals during the pandemic. On the other hand, Swedish registry, French registry, and a German study by Primessnig et al. showed no difference in patients’ characteristics such as age, gender, and prevalence of risk factors between pre-pandemic and pandemic period [12-14].

## Conclusions

In conclusion, venous and arterial thrombosis are frequently associated with COVID-19 infection and cardiovascular complications are commonly seen in patients with COVID-19. Our case report is unique based on the fact that the patient had significant thrombosis and the proximal lesion was the source of thromboembolism resulting in occlusion of the peripheral vessels and required prolonged intravenous tirofiban infusion. The patient responded well to tirofiban infusion and did not require immediate percutaneous coronary intervention. The learning point from this case is that prolonged duration treatment of tirofiban should be offered to such patient as immediate stenting will increase the risk of in-stent restenosis and loss of reperfusion.
